# Designing a Novel Hybrid Technique Based on Enhanced Performance Wideband Millimeter-Wave Antenna for Short-Range Communication

**DOI:** 10.3390/s24103219

**Published:** 2024-05-18

**Authors:** Tanvir Islam, Dildar Hussain, Fahad N. Alsunaydih, Fahd Alsaleem, Khaled Alhassoon

**Affiliations:** 1 Department of Electrical and Computer Engineering, University of Houston, Houston, TX 77204, USA; tislam7@cougarnet.uh.edu; 2 Department of Data Science, Sejong University, Seoul 05006, Republic of Korea; hussain.bangash@sejong.ac.kr; 3 Department of Electrical Engineering, College of Engineering, Qassim University, Buraydah 52571, Saudi Arabia; f.alsunaydih@qu.edu.sa (F.N.A.); f.alsaleem@qu.edu.sa (F.A.)

**Keywords:** coupling reduction, MIMO antenna, millimeter-wave, wideband, 28 GHz

## Abstract

This paper presents the design of a performance-improved 4-port multiple-input–multiple-output (MIMO) antenna proposed for millimeter-wave applications, especially for short-range communication systems. The antenna exhibits compact size, simplified geometry, and low profile along with wide bandwidth, high gain, low coupling, and a low Envelope Correlation Coefficient (ECC). Initially, a single-element antenna was designed by the integration of rectangular and circular patch antennas with slots. The antenna is superimposed on a Roger RT/Duroid 6002 with total dimensions of 17 × 12 × 1.52 mm^3^. Afterward, a MIMO configuration is formed along with a novel decoupling structure comprising a parasitic patch and a Defected Ground Structure (DGS). The parasitic patch is made up of strip lines with a rectangular box in the center, which is filled with circular rings. On the other side, the DGS is made by a combination of etched slots, resulting in separate ground areas behind each MIMO element. The proposed structure not only reduces coupling from −17.25 to −44 dB but also improves gain from 9.25 to 11.9 dBi while improving the bandwidth from 26.5–30.5 GHz to 25.5–30.5 GHz. Moreover, the MIMO antenna offers good performance while offering strong MIMO performance parameters, including ECC, diversity gain (DG), channel capacity loss (CCL), and mean effective gain (MEG). Furthermore, a state-of-the-art comparison is provided that results in the overperforming results of the proposed antenna system as compared to already published work. The antenna prototype is also fabricated and tested to verify software-generated results obtained from the electromagnetic (EM) tool HFSS.

## 1. Introduction

Multiple-input–multiple-output (MIMO) antenna systems have gained a lot of attention owing to their ability to expand the capacity and reliability of wireless communication systems [1]. The rectification of the devices is undertaken to obtain performances according to the needs of 5G and future 6G communication systems [2]. Modern-day devices require a high data rate and high-speed coverage along with the rising challenge of size constraint, which results in a huge demand for low-profile and compact RF components [3,4]. These changes in communication models force the designer to further revise the requirements of antenna design. For MIMO antennas, mutual coupling between the antennas can considerably affect the outcomes of MIMO systems [5]. Therefore, there is a dire need to develop techniques to lower the coupling among MIMO elements and consequently improve the performance of MIMO systems [6].

In order to minimize the amount of mutual coupling in MIMO antenna networks, a number of approaches have been advised, including employing metamaterials via pins, parasitic elements, and defective ground structures (DGS) [7,8,9]. In order to alter the antenna’s electromagnetic properties and improve isolation among the antennas, parasitic structures are passive components added to the antenna design. It has been demonstrated that using parasitic elements can improve the isolation of MIMO antennas, and numerous designs employing parasitic elements have been suggested to improve the functionality of MIMO antennas [10,11]. Conventionally, filters can be utilized to suppress the interference of the signal among single device elements [12,13]. However, adding filters in the circuit introduces complexity along with the requirement of additional matching circuits that require a large physical area [14,15]. Surface waves are attenuated, and DGS are incorporated into the ground plane of the antenna to lessen coupling between the antennas [16]. DGS can also increase an antenna’s bandwidth and gain while lowering its emission in undesirable directions [17].

In addition to the isolation, the bandwidth, peak gain, size, and geometrical configuration are also important parameters to analyze while studying and designing MIMO antenna systems for future communication systems. The high value of peak gain can be received by using a phased array technique, as used in [18], but the setbacks of a high profile or large dimension and its complexity due to the array cause troubles in system integration. On the other hand, the compact antenna in [19] has a dense size and simplified construction, but it also has a narrow operational bandwidth as well as a small value gain. An antenna with a dense size and structure geometry, which offers wideband and high peak gain, is challenging [20].

In [21], a phased array antenna functional over a wideband of 26.5–29.5 GHz and a high value of gain 11.9 dBi was revealed for 5G millimeter-wave implementations. The antenna operates over high gain and wideband but has the demerit of complex geometry. One more design, again with a high profile and complexity for 28 GHz applications, offers a wideband of 3 GHz with a moderate value gain, is reported in [22]. It can be concluded from the literature that single-element antennas reported for 28 GHz applications either have high gain, wideband, complex geometry, and large size [23] or dense size and simplified geometry with a narrow band and low value of gain [24,25].

As stated in the previous paragraph, MIMO systems are implemented to boost the ability of antennas to operate at high data rates and reduce latency in the system [26]. For mm-wave applications, a twin port MIMO antenna with a substrate-integrated waveguide (SIW) feed was reported in [27]. The antenna features an air-filled slot and a high peak gain of about 6.9 dBi, although it only has a small bandwidth of 0.4 GHz. For 5G applications, a further antenna with a bandwidth of 26.5–29.5 GHz is mentioned in [28]. The antenna provides a gain of 7.1 dBi and 30 dB isolation. Reference [29] presents a small antenna that operates at 28.2–30.7 GHz and has an overall dimension of 10 mm × 12 mm. Although the antenna is small, it uses a frequency range that is not globally assigned.

A four-port MIMO antenna with DGS ground architecture is provided for 5G wideband components in [30]. The active frequency range of the antenna is 25.25–29.5 GHz, and its general dimensions are 30 × 35 × 0.76 mm^3^. The antenna operates over a wideband with strong gain, has a complex shape, and is small in size. Another wideband, high-gain antenna is attested to in [31]. The antenna has a bandwidth of 23–40 GHz with a peak gain of 12 dBi. The antenna is large, 80 mm × 80 mm × 1.57 mm. Reference [32] offers a millimeter-wave application-specific wideband circularly-polarized (CP) magneto-electric dipole antenna. With a peak gain of 8.5 dBi, the antenna works over a wideband of 24.2–31.8 GHz.

In order to investigate the characteristics of the MIMO antenna system, the MIMO antenna must be isolated [33,34,35,36,37,38,39,40,41,42,43,44]. Isolation, often referred to as mutual coupling, is a measurement or analysis of the influence of one MIMO antenna system component over another. In order to increase the isolation to 24 dB, metal strips are used in dielectric resonator antennas [33]. The aforementioned device works over a restricted range from 27.5 to 28.35 GHz and is only 20 mm × 20 mm in size. Each antenna element has a C-shaped parasitic patch overlaid over it to mitigate mutual interactions among MIMO elements. Antenna isolation is upgraded from 17 to 32.32 dB. The antenna has a compact size of 15 mm × 26 mm but operates over a narrow bandwidth of 1.5 GHz and offers a low value of the envelope correlation coefficient (ECC) = 0.14 [34].

The isolation between MIMO aerial elements is improved to 25 dB in [35] by employing a DGS ground plane. The antenna has a complicated layout and works at a frequency range from 27 to 30.5 GHz with a compact footprint of 25 × 15 × 1 mm^3^. Reference [36] described a separate proposal that makes use of a DGS ground plane to boost isolation. The antenna’s working bandwidth is 27.5–28.5 GHz, and its lowest coupling is observed to be −40 dB. A metamaterial-based aerial for isolation improvement was provided in [37]. The antenna’s compact size is 26.5 mm by 14.5 mm, and its operational bandwidth is from 26.5 to 30 GHz. To achieve 39 dB of isolation, the metamaterial structure is loaded. Another antenna with a geometry of 20 mm × 40 mm with an operational bandwidth of 26.7–29.7 GHz, having a maximum isolation of 29 dB after loading metamaterial, is reported in [38]. This design has the setbacks of complex geometry and difficulty in integrating with other devices.

In [39], a slotted stub-loaded ground plane was utilized to refine the isolation of the Ultra-wideband (UWB) antenna to −22 dB. Width is provided by an antenna with an ECC value of 0.15, and it measures 33 mm × 48 mm × 1.6 mm. The antenna has a simplified structure, but the low value of isolation is improved as it may be crucial for high data devices. The isolation of the antenna is reduced by electronic bandgap (EBG) in [40]. The antenna features a modest ECC value of 0.0015, a minimal isolation of roughly −21 dB, and an ultimate measurement of 48 mm × 31 mm × 0.254 mm. It features broad operation between 26 and 31 GHz and a decent ECC value, but it also has a complex geometry and poor minimum isolation. Reference [41] presented another small antenna with overall dimensions of 41 mm × 28 mm × 0.787 mm. A minimum isolation of −37 dB is offered by the antenna, but it has a narrow bandwidth of around 1.9 GHz (24.6–26.5 GHz).

A wideband antenna operating over 3.1–10.6 GHz for 5G applications is reported in [42]. The antenna isolation is improved to > −20 by using DGS. The antenna is unable to improve much isolation and has a complex geometry. Another wideband antenna operating over 27–32 GHz offers isolation of −39 dB by using meta surface [44]. The antenna has setbacks due to its large size and complex geometry.

The review of work presented in the literature for 28 GHz applications and MIMO antenna systems shows the adoption of various techniques for isolation improvement has been studied. It is clear from the discussion that there is still a gap in research to build and construct antennas having small and simple geometry, wideband, high gain, and low coupling with good value of other MIMO parameters, such as ECC, diversity gain (DG), channel capacity loss (CCL), etc. In this article, a MIMO antenna with DGS and parasitic patch is designed to refine the isolation, gain, and bandwidth of the antenna. The antenna is small in dimension, has a simple geometrical configuration, and offers wideband, high gain, and low mutual coupling.

## 2. Design Method of Proposed Antenna

### 2.1. Single Element Design

**Figure 1:**
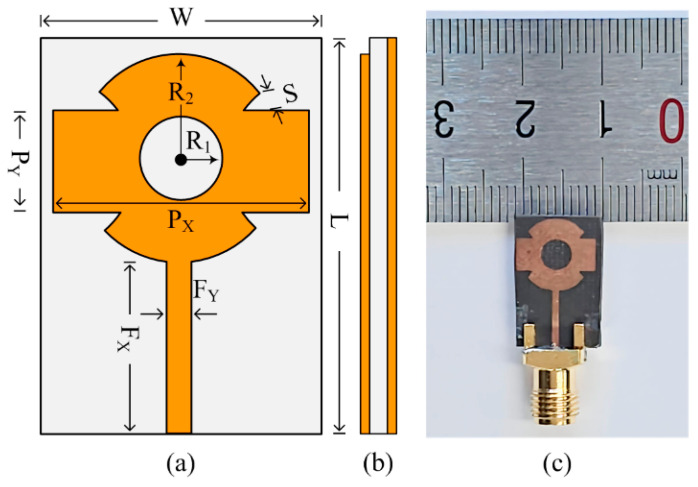
Structure of suggested antenna. (**a**) Top view, (**b**) side-view, (**c**) hardware prototype.

Figure 1 depicts the geometrical construction of the presented antenna design along with its fabricated prototype. The antenna was constructed using rectangular and circular patches with circular-shaped slots in the center. The radiating patch was connected with a microstrip feedline with a quarter wave matching of 50 Ω, full ground plane, and placed on the top of substrate material Roger 6002. The substrate material used is available in the market with a specification of relative permittivity of Ԑr = 2.2 and loss tangent tanδ = 0.0009 with a thickness of T = 1.52 mm. The single-element design had a compact dimension of W × L = 12 mm × 17 mm. The optimized parameter of the proposed design is given below:

*W* = 12; *L* = 17; *P_X_* = 10; *P_Y_* = 4; *F_X_* = 7.5; *F_Y_* = 0.75; *S* = 1; *R*_1_ = 2; *R*_2_ = 4.5. All units are in millimeters (mm).

### 2.2. Design Stages to Construct Single Element

The final geometry with the required results was obtained after following four main design steps.

**Figure 2:**
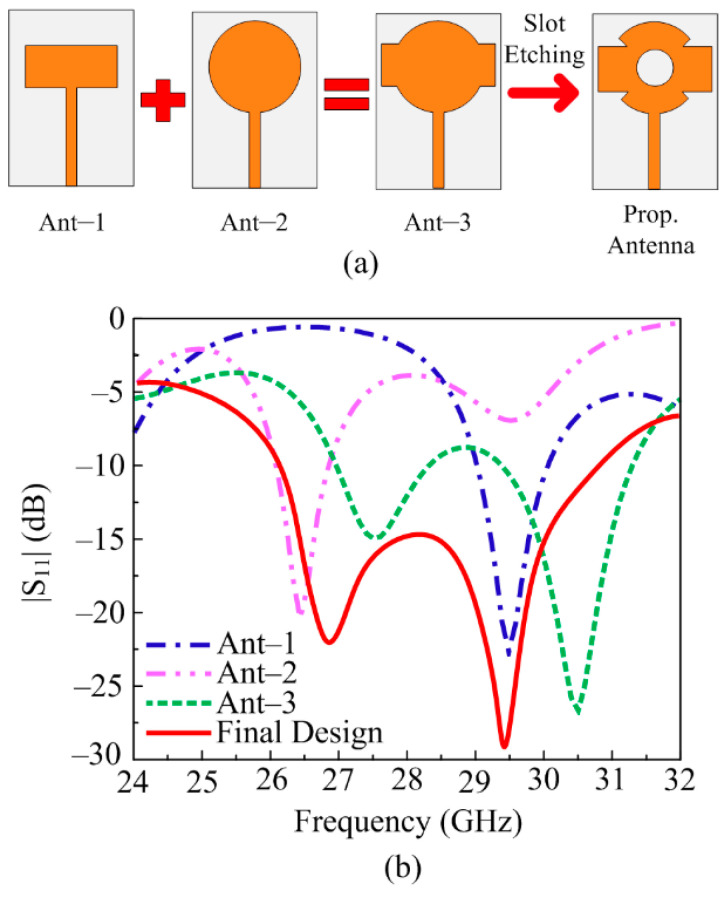
(**a**) Design stages of suggested antenna, (**b**) impact of design stages on |S_11_|.

Step 1: Designing a rectangular patch antenna. In the beginning, the patch antenna formula described in [45], as well as given in Table 1, is used for generating a patch with a rectangular shape antenna. The antenna has a rectangular radiator with dimensions of *P_X_* × *P_Y_* = 10 mm × 4 mm; the antenna operates over 28.9–30.5 GHz with S_11_ of <−20 dB as given in Figure 2a,b. The following Equations (1)–(3) are utilized to find the initial value of the patch:

(1) $$\mathrm{Leff}=\frac{c}{2F\sqrt{Ԑreff}}$$

(2) $$\text{Ԑ}\mathrm{reff}=\frac{Ԑr+1}{2}+\frac{Ԑr-1}{2}\left[\frac{1}{1+12\frac{H}{W}}\right]$$

(3) $$\Delta L=0.412H\;\frac{\left(Ԑreff+0.3\right)(\frac{W}{H}+0.264)}{\left(Ԑreff-0.258)(\frac{W}{H}+0.8\right)}$$

Step 2: Designing circular patch antenna. Then, using the formula given in [46] and Table 1, a circular patch antenna is created. The antenna has a circular radiator with a radius of R_2_ = 4.5 mm; the antenna operates over 26–27.2 GHz with S_11_ of <−18 dB, as mentioned in Figure 2b. The radius of the radiator can be estimated using the following Equations (4) and (5):

(4) $$\mathrm{Reff}=R\left\{\sqrt{1+\frac{2H}{\pi ԐrR}\left(ln\frac{\pi R}{2H}+1.7726\right)}\right\}$$

(5) $$F=\frac{1.8412\times c\;}{4\pi \mathrm{Reff}\sqrt{\text{Ԑ}r\;}}$$

Step 3: Merging rectangular and circular patch antenna. In this step, the rectangular and circular antennas designed for millimeter-wave applications are merged. The resultant antenna obtained is given in Figure 2. The resultant design offers a wideband of 27–31.5 GHz with S_11_ of <−22 dB, as shown in Figure 2b. Lowering the value of S_11_ is required as in the middle of the operational bandwidth, the plot is not much below −10 dB.

Step 4: Slots insertion from the antenna. To improve the return loss for wideband operation, the circular-shaped slot, having radius R1 = 2 mm, is etched from the center of the radiating patch, along with a small alteration at the edges where the circular patch and rectangular patch meet. These slot insertion steps help to reduce the value of S_11_, and the final design is obtained with an operational bandwidth of 26.1–31.7 GHz.

### 2.3. Results of Single Element

**Figure 3:**
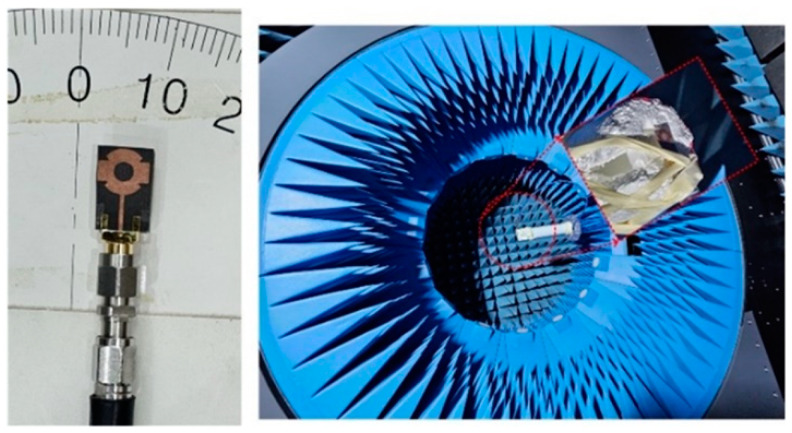
Measurement setup for studying design parameters of the suggested single element of the antenna.

Various design parameters in the form of |S_11_|, and gain and radiation patterns were studied to endorse the functionality of the antenna for 5G applications. The high frequency structure simulator (HFSS) software tool was used to conduct the modeling inquiry, and a physical model was developed, as well for evaluating the software’s findings. Figure 3 shows the testing arrangement for the prototype antenna, which used a vector network analyzer (VNA) to determine the |S_11_| parameter in open air. The anechoic chamber was employed to evaluate far-field characteristics. The horn antenna was utilized as a form of transmitter and the recommended antenna as a receiver in an anechoic chamber.

#### 2.3.1. S-Parameter and Gain

**Figure 4:**
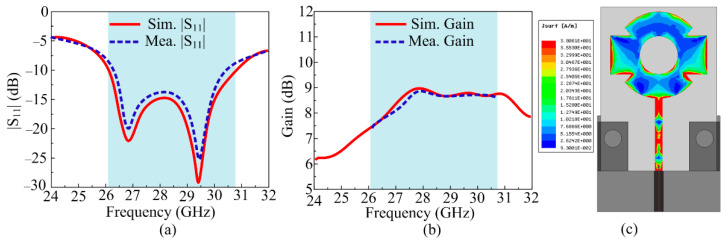
(**a**) S-Parameter and (**b**) gain of the suggested antenna. (**c**) Current analysis at 28 GHz.

The S-parameter graph of both the prototype tested and the model simulated was generated by the design that is recommended in Figure 4a. The illustration shows that the schematic provided an impedance bandwidth (|S_11_| < −10 dB) of 26.1–30.7 GHz, with resonance frequencies of 26.5 GHz and 29.5 GHz. The outcomes of the equipment test and the software results indicated an elevated level of consistency. Figure 4b shows the gain versus frequency plot of the suggested antenna. With a peak gain of 9 dBi at roughly 27 GHz, the recommended antenna provided a gain of >8 dBi over the operational bandwidth of 26.1–30.7 GHz. Between simulated and tested findings, there was a notable degree of consistency. The value of gain and bandwidth offered by the suggested work make it a capable and best applicant for future 5G devices. The current distribution of the proposed antenna at 28 GHz is given in Figure 4c. It can be seen that the current was fairly distributed across the radiator, which resulted in a symmetrical radiation pattern across the *X*-axis.

#### 2.3.2. Radiation Pattern

**Figure 5:**
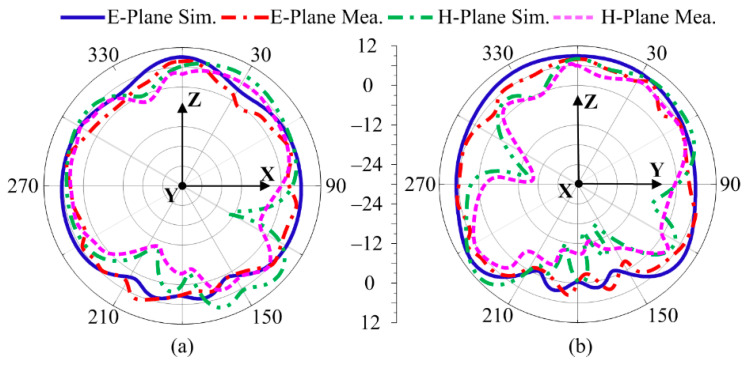
Radiation pattern. (**a**) 28 GHz, (**b**) 30 GHz.

Figure 5a,b display the computed and hardware prototype-tested radiation pattern of the designed antenna for particular frequencies of 28 and 30 GHz. According to the supporting figure, the aerial had a regular shape with a low back and level side lobes. The antenna radiated over a broadside pattern in the E-plane (θ = 0°) and H-plane (θ = 90°) at both operational frequencies.

Also, a contrast between the recommended wideband antenna design and work published in the past was made for the 28 GHz 5G application. Table 1 shows that the suggested design was either wideband and had a high gain in comparison to other studies or small and simple in geometry. As a result, the findings and comparison with the literature indicated that the recommended approach is the best candidate for devices operating on 28 GHz.

## 3. Designing MIMO Configuration of Antenna with Novel Hybrid Technique

**Figure 6:**
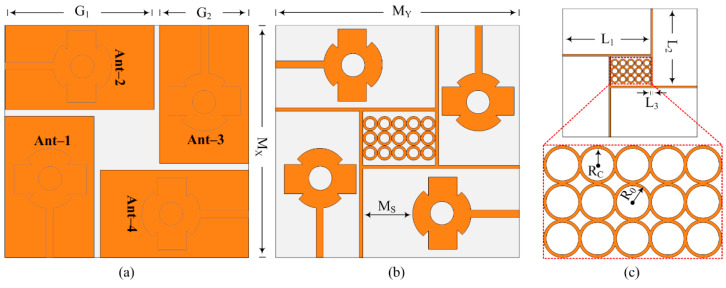
Geometrical structure of suggested DGS and parasitic patch loaded MIMO antenna. (**a**) Back view, (**b**) front view, (**c**) parasitic patch.

**Figure 7:**
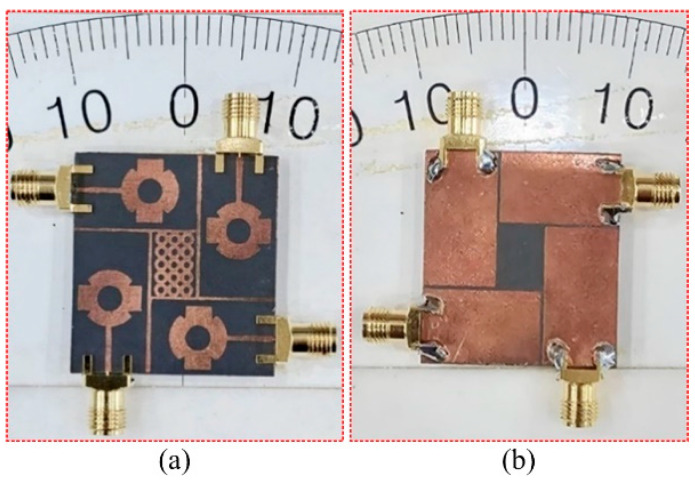
Hardware prototype of suggested MIMO antenna, (**a**) Top view, (**b**) back view.

The structural configuration of the suggested 4-port (2 × 2) MIMO antenna is given in Figure 6a–c. The suggested MIMO design was derived from a four single-element antenna structure placed orthogonally on the top of a single substrate. The spacing between two nearby MIMO elements was MS = 9 mm. The hybrid decoupling structure, which consisted of novel DGS and a parasitic patch, was placed between MIMO elements. The novel DGS ground plane reduced the mutual coupling as well as purifying the bandwidth and gain of the antenna, whereas the suggested structure of the parasitic patch also helped in improvement in bandwidth and gain, along with a refining of isolation. The novel parasitic patch contained a line strip and rectangular box structure filled with a 5 × 3 array of circular rings. The overall effect of both DGS and a parasitic patch significantly improved bandwidth and gain with a huge increase in isolation between MIMO elements. A snap of the hardware prototype is given in Figure 7a,b. The optimized parameters of the suggested MIMO design are shown as:

*M_X_* = 35; *M_Y_* = 32; *M_S_* = 9; *G*_1_ = 12; *G*_2_ = 19.5; *L*_1_ = 22.5; *L*_2_ = 12; *L*_3_ = 0.5; *R_C_* = 0.5; *R*_0_ = 1. (All units are in mm).

### 3.1. Design Procedure of MIMO Antenna

#### 3.1.1. Orthogonally Placed Element MIMO Antenna

**Figure 8:**
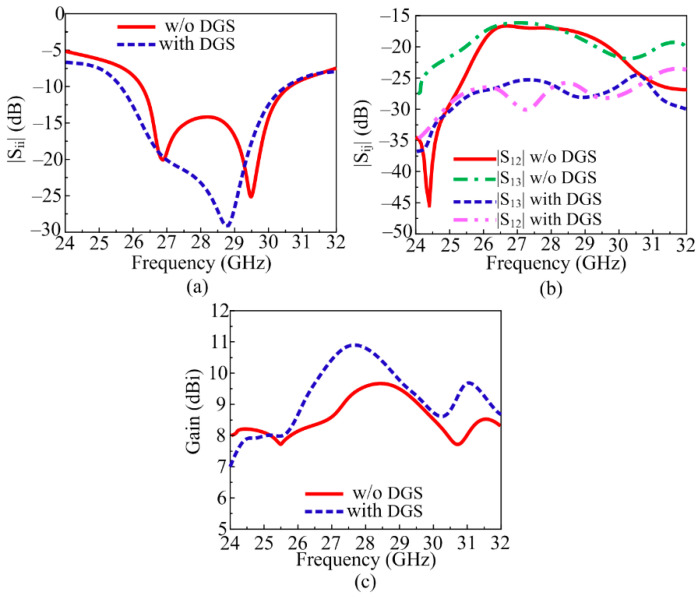
Comparison between simple MIMO antenna and MIMO antenna with DGS. (**a**) Reflection coefficient, (**b**) transmission coefficient, (**c**) simulated gain.

Four antennas were arranged orthogonally in the preferred MIMO antenna. The features of the MIMO antenna were identical to those of the single element described in the section above, but because four antennas were loaded onto the substrate without any decoupling structures, the size of the substrate increased. The aerial measured *M_X_ × M_Y_* = 37 mm × 32 mm overall. Without DGS and a parasitic patch, the aforementioned antenna provided a wideband of 26.5–30.5 GHz with a peak gain of 9 dBi at roughly 30 GHz. The antenna’s performance demonstrated the importance of separation between antenna parts. According to Figure 8, the mutual coupling reported from such a configuration was around −17.5 dBi (c). An additive white Gaussian noise (AWGN) channel SNR of 30 dB was necessary for rapid data transfer and noise-free communications. To meet these requirements, isolation should be under −30 dB.

#### 3.1.2. MIMO Antenna with DGS

As in the literature review, various techniques were used to minimize mutual coupling, one of which was introducing DGS. This fact has also been seen in literature studies: defected ground structure (DGS) also helps in the improvement in bandwidth and gain. The novel DGS was employed in the suggested MIMO antenna to not only refine mutual coupling but also upgrade the gain and bandwidth. The novel DGS was obtained by etching the rectangular slot from the center and the strip line slot out of the copper layer of the ground plane. The results in Figure 8a–c show the comparison between results obtained from the simple MIMO antenna and the MIMO antenna with DGS. It can be examined from the figures that the antenna with DGS offered a wide bandwidth of 25.5–30.3 GHz. The value of the gain of the suggested MIMO antenna was also improved from 9.25 to 10.9 dBi, as given in Figure 8c. The mutual coupling, which is a key parameter to analyze, was improved from −17.25 to −27.5 dB. The transmission coefficient between the adjacent antenna (|S_12_|, |S_23_|, |S_34_|, |S_41_|), as well as the diagonal element (|S_13_|, |S_24_|), are given in Figure 8b.

#### 3.1.3. MIMO Antenna with Decoupling Patch

**Figure 9:**
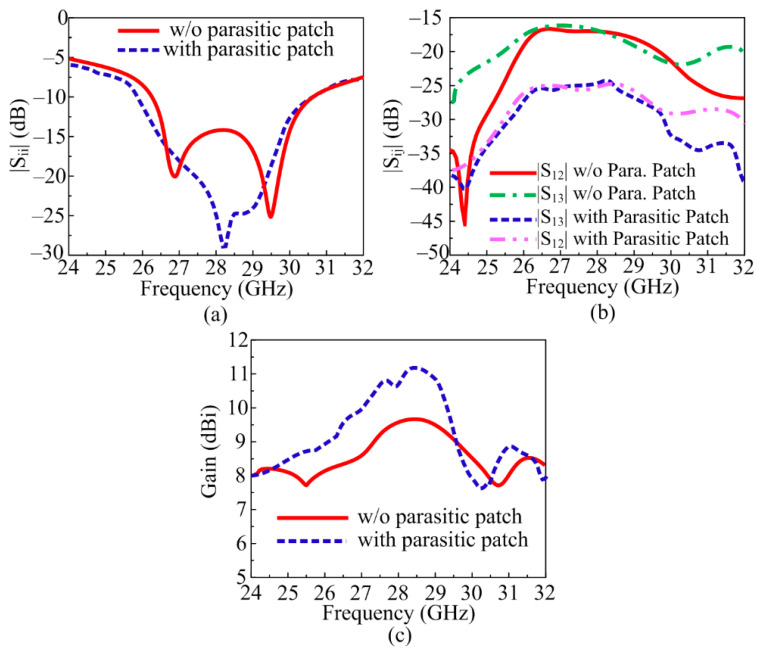
Comparison between simple MIMO antenna and MIMO antenna with Parasitic patch. (**a**) Reflection coefficient, (**b**) transmission coefficient, (**c**) simulated gain.

In the literature study, it was studied and discussed that an antenna loaded with a parasitic patch helps to refine the isolation of the MIMO antenna system. A unique parasitic patch was positioned between the MIMO antenna elements of the designed MIMO antenna. The strip line and 5 × 3 rings found in the newly identified parasitic element are shown in Figure 6b. The dimension of the suggested parasitic element was fixed to obtain the best results. After loading the parasitic patch between MIMO antenna elements, not only was mutual coupling reduced, but bandwidth and gain also improved. It can be observed from Figure 9a that the bandwidth was improved and S_11_ reduced. The designed MIMO antenna with a parasitic patch had an S_11_ of −29 dB and ran between 25.5 and 30.1 GHz. As shown in Figure 9b, the isolation of an antenna filled with a decoupling structure increased from −17.25 dB to 28 dB. The comparison of gain provided in the figures shows that antenna gain was improved from 9.25 dBi to 11 dBi, as given in Figure 9c.

#### 3.1.4. MIMO Antenna with Novel Hybrid Technique

**Figure 10:**
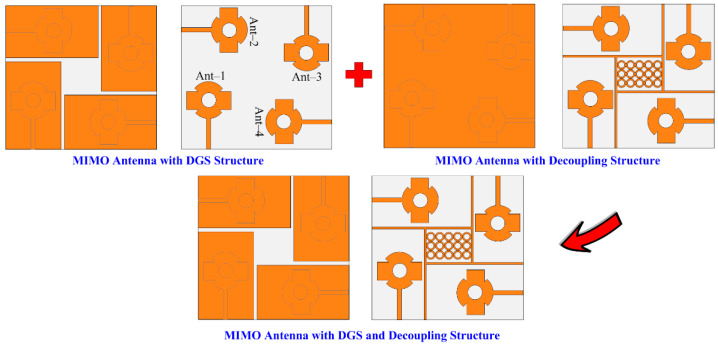
Design stages of suggested MIMO antenna with hybrid technique.

**Figure 11:**
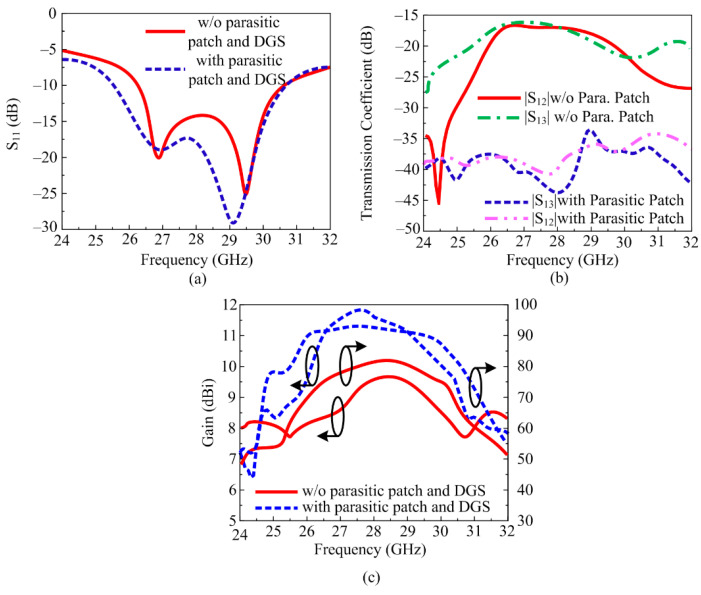
Comparison between simple MIMO antenna and MIMO antenna with the hybrid technique. (**a**) Reflection coefficient, (**b**) transmission coefficient, (**c**) gain and efficiency.

The analysis provided above makes it obvious that antennas with parasitic patches and DGS delivered upgraded bandwidth, gain, and isolation. The suggested layout of the antenna, shown in Figure 10, was created by combining the MIMO antenna with DGS and the MIMO antenna with a parasitic patch. The MIMO antenna packed with hybrid architecture (DGS and parasitic patch) gave a wideband of 25.5–30.5 GHz with a low S_11_ value of −30 dB, as can be demonstrated in Figure 11a. As shown in Figure 11b, the antenna’s isolation increased from 17.25 dB to 44 dB. And the MIMO antenna with DGS and parasitic patch had a gain of 11.9 dBi, as given in Figure 11c. In Figure 11, a juxtaposition between a MIMO antenna loaded with a parasitic patch and DGS and one without DGS is shown. The efficiency of the MIMO antenna was also presented, and a 20% improvement was observed.

After loading the parasitic element into the proposed four-port MIMO antenna, the power from the excited patch behaved toward the parasitic patches rather than the non-excited patches, which resulted in an improvement in the isolation of the MIMO antenna. The coupling between elements had slight differences, which depended on the shape, structure, and number of parasitic patches. The gain of the proposed MIMO antenna was improved by loading parasitic patches due to a larger radiating aperture.

In Table 2, the comparison of parameters in terms of bandwidth, gain, and isolation for all four cases is given. It can be seen that the antenna offered good values of gain, bandwidth, and low coupling when both a parasitic patch and DGS were loaded.

## 4. Results and Discussion

**Figure 12:**
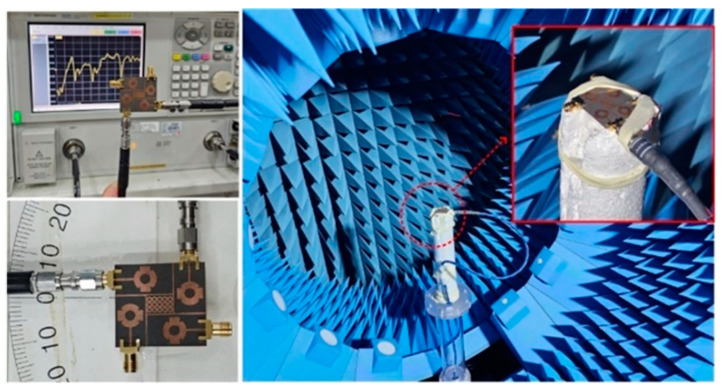
Snaps of measurement setup of the suggested MIMO Antenna.

For the verification of the software-generated results and suggested concept, the hardware of the design was fabricated. The fabricated prototype was examined by utilizing a vector network analyzer (VNA) of model N5224A for the near-field S-parameter verification and an anechoic chamber for the far-field measurements, as given in Figure 12. The large similarity between the tested and software-generated results was observed, which shows the potential of suggested antenna. The little difference may be due to imperfections in the measurement framework or fabrication tolerance.

### 4.1. S-Parameter

**Figure 13:**
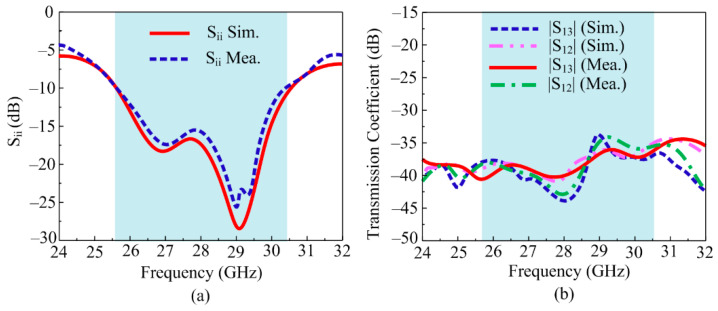
Comparison between the simulated and tested (**a**) reflection and (**b**) transmission coefficients.

In Figure 13, the contrast between simulated and tested S-parameters in terms of reflection and transmission coefficients is given. It can be noted from Figure 13a that the suggested parasitic patch and DGS-loaded 4-port MIMO antenna give wide impedance bandwidth (|S11| < −10), ranging from 25.6 to 30.4 GHz for millimeter-wave applications. Across the band, for the mutual coupling of the suggested antenna, as demonstrated in Figure 13b, the antenna had a mutual coupling of <−35 dB for the orthogonal element, whereas the mutual coupling was <−44 dB for the adjacent element. The offered value of the mutual coupling was less than −20 dB, which is under the acceptable range. Moreover, the similarity in tested and software-generated outcomes is also observed from the figures. The resemblance in tested and software results and operational values of reflection and transmission coefficients cause the suggested antenna to be a dominant applicant for future 5G devices.

### 4.2. Radiation Pattern

**Figure 14:**
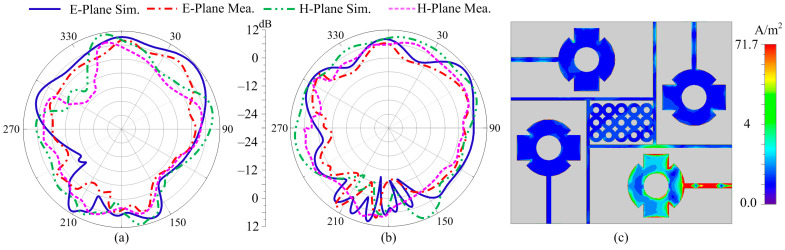
Radiation pattern of suggested antenna at (**a**) 28 GHz (**b**) 30 GHz, (**c**) current distribution at 28 GHz.

Figure 14a,b shows the preferred MIMO antenna’s tested and software-generated radiation pattern, which included a parasitic patch and a DGS. The radiation pattern at the targeted frequencies of 28 and 30 GHz was examined. The figures show that the antenna delivered a constant pattern with low back and level side lobes. For both operational frequencies, the aerial radiated over a broadside pattern in the E-plane (θ = 0°) and H-plane (θ = 90°). Additionally, the suggested MIMO antenna design’s resemblance of the tested and predicated patterns revealed a striking similarity, making the antenna a great candidate for upcoming 5G applications. Furthermore, the current analysis in Figure 14c revealed the effectiveness of the decoupling structure, which stops the current from being induced in other elements, resulting in low coupling.

### 4.3. Gain

**Figure 15:**
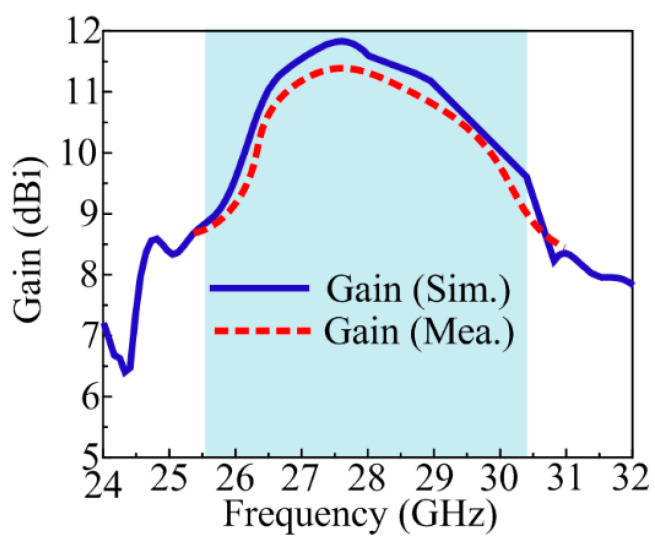
Gain verse frequency plot of suggested antenna.

Figure 15 depicts the simulated and hardware-tested gain-versus-frequency curve of the suggested MIMO antenna. It can be noticed from the figure below that the preferred MIMO antenna with DGS and parasitic patch offers a large value gain over the operational region. The antenna offers gain > 9 dBi over the operational band of 25.5–30.5 GHz with a peak value of 11.9 dBi around 28 GHz. Moreover, the strong resemblance between tested and simulated gain makes the suggested antenna a viable candidate for high-gain devices using 5G in the near future.

### 4.4. Envelop Correlation Coefficient

The validation of the performance of each element of the MIMO antenna was examined by studying the envelope correlation coefficient (ECC), which can also be calculated by the equation below [47].

(6) $$\left|\rho_{e}\right|=\frac{|\iint_{4\Pi }{\left[\vec{{F^{*}}_{1}}\left(\theta ,\Phi \right)\;\vec{F_{2}}\left(\theta ,\Phi \right)u]\right|}^{2}}{\iint_{4\Pi }|{\vec{F_{1}}\left(\theta ,\Phi \right)|}^{2}\;d\Omega \iint_{4\Pi }|\;{\vec{F_{2}}\left(\theta ,\Phi \right)|}^{2}d\Omega }$$

**Figure 16:**
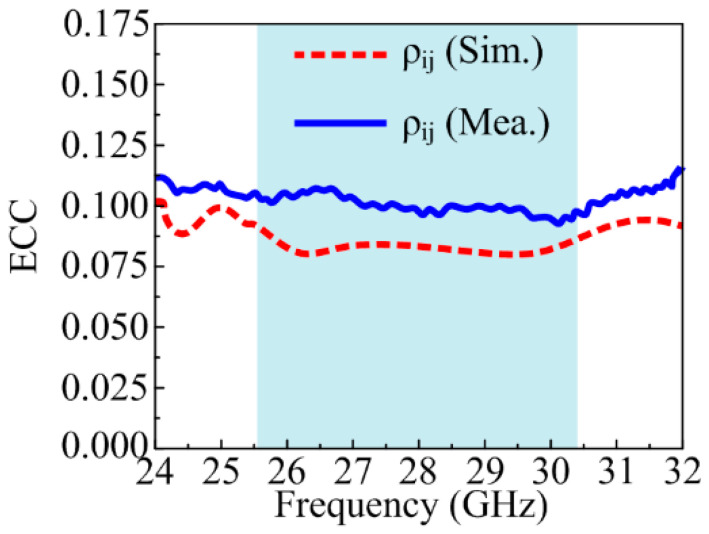
ECC of suggested MIMO antenna.

The ECC of the suggested antenna was calculated with respect to electric fields. $\vec{F_{1}}\left(\theta ,\Phi \right)$ represents the first vector field, while the second vector field is represented by $\vec{F_{2}}\left(\theta ,\Phi \right)$. Moreover, $\vec{{F^{*}}_{1}}\left(\theta ,\Phi \right)$ represents the complex conjugate of the first electric field. The simulated and measured results illustrated in Figure 16 show that the proposed antenna offers a low ECC < 0.12 in the band of interest. The ECC value will result in high spectral efficiency along with high reliability and data rates.

### 4.5. Diversity Gain

During transmission in the MIMO antenna, some power losses occur, which can be analyzed by studying diversity gain (DG). The numerical value of DG can be calculated using the equation below. The ideal value of DG for any MIMO is around 10 dB [48].

(7) $$DG=10\;\sqrt{1-|{ECC|}^{2}}$$

**Figure 17:**
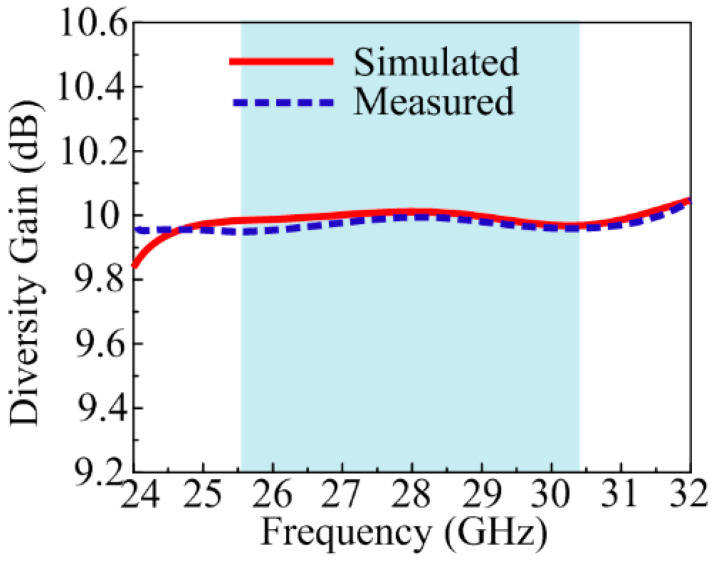
Diversity gain of suggested MIMO antenna.

Figure 17 shows the DG of the suggested antenna. The figure indicates that the suggested design provided a DG of around 9.99 dB through all the operational bands.

### 4.6. Channel Capacity Loss

The value of the channel loss that occurs and permits a signal message to transmit over any communication channel was measured by studying channel capacity loss (CLL). The ideal value of CLL should not be greater than 0.4 bits/s/Hz, but a value that approximately approaches this range is to be considered good. The mathematical relation to calculate CCL is also given below [49].

(8) $$C_{Loss}=-{log}_{2}\det(\propto^{R})$$

**Figure 18:**
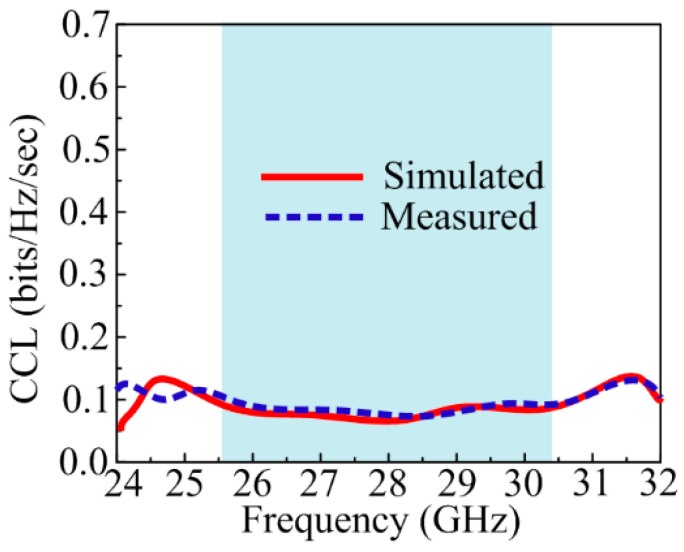
CCL of suggested MIMO antenna.

Figure 18 shows the CLL of the suggested parasitic patch and DGS-loaded MIMO antenna. The suggested design offers a CLL of around 0.1 bits/s/Hz through all operational bands.

### 4.7. Mean Effective Gain

Mean effective gain (MEG), which is the ratio of incident and received power, is another crucial MIMO metric. This parameter’s allowable range should be from −3 dB to −10 dB. Also included below [50] is the mathematical relationship used to calculate the MEG.

(9) $${MEG}_{i}=\frac{P_{rec}}{P_{inc}}=\oint \left[\frac{XPR.\;G_{\theta i}\left(\Omega \right)+G_{\emptyset i}\left(\Omega \right).P_{\emptyset }\left(\Omega \right)}{1+XPR}\right]\;d\Omega$$

**Figure 19:**
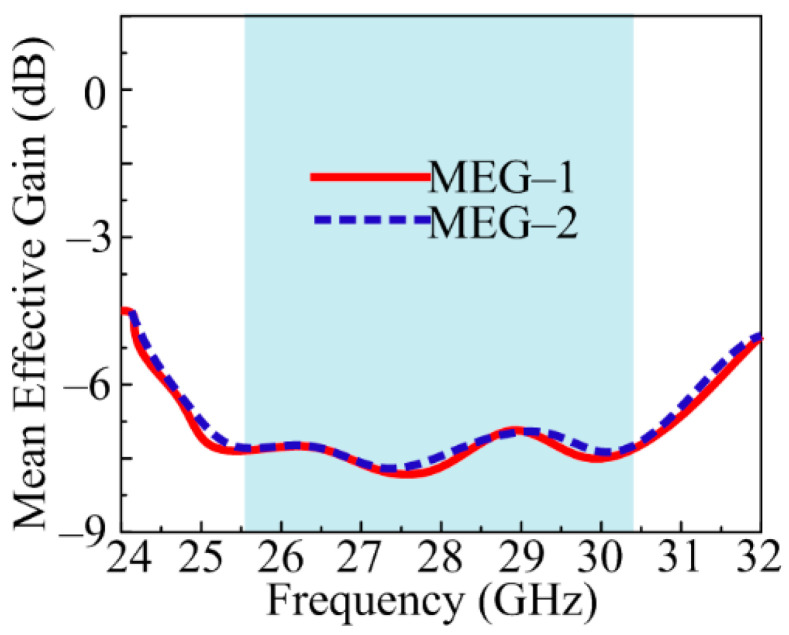
Mean effective gain of suggested MIMO antenna.

The MIMO antenna proposed in this work offers an MEG of around −8 dB. The offered value is between the acceptable range, as shown in Figure 19.

### 4.8. Antenna with a Model Car

**Figure 20:**
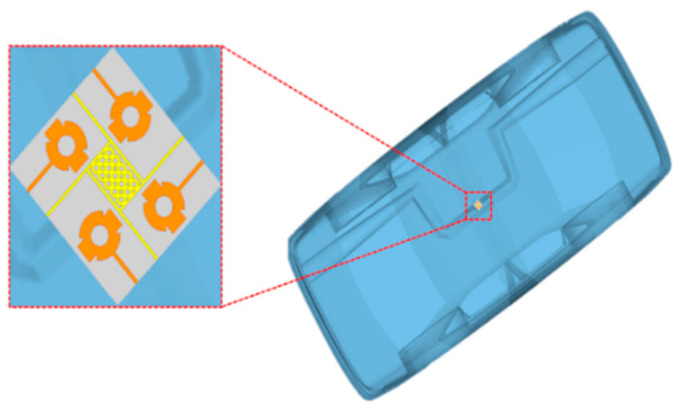
Simulated setup for V2X analysis.

**Figure 21:**
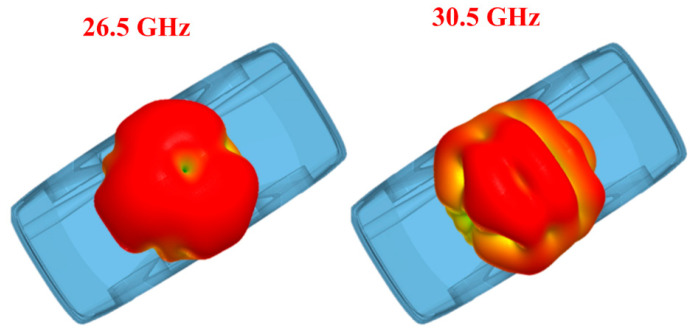
Radiation pattern offered by the suggested antenna.

The suggested antenna was simulated over a model car to verify its practical usage for V2X (vehicle to everything) communications by considering the accuracy of the location as described in [51,52]. The DGS is very sensitive to the ground plane, so this analysis validated the antenna performance over car models for practical applications. The suggested antenna was placed 6 mm above the roof of the car, which is usable for almost all practical applications [53,54], as given in Figure 20. The radiation pattern, shown in Figure 21, represents the identical behavior of the antenna over the model car. The 3-D pattern of the radiation behavior in the figure below was analyzed when port 1 was excited.

### 4.9. Comparison with the Literature

The comparison of the proposed MIMO antenna with related works is shown in Table 3. It can be seen that the proposed antenna offers compact physical as well as compact electrical size as compared to the rest of the antennas, except for the work reported in [43]. However, the MIMO antenna reported in [43] offers a narrow bandwidth that is not suitable for global applications. Moreover, the source of measuring ECC in the reference work is S-parameters, which are not reliable and always offer very low ECC due to the smaller value of S-parameters. Contrary to them, electric fields were used to calculate ECC in the proposed work, and the results offered a value of less than 0.12. Thus, it can be concluded that the proposed work presents a good combination of compact size, broadband, and low ECC along with gain improvement while incorporating a geometrically simple structure.

## 5. Conclusions

A wideband and high gain antenna operating at 28 GHz was designed and presented for millimeter-wave applications. The MIMO antenna was derived from single-element antennas in order to enhance its performance in terms of gain, bandwidth, and coupling. The basic MIMO antenna has the demerit of high coupling around −17.25 dB, which is not acceptable for mm-wave systems. To reduce mutual coupling, a novel hybrid technique, which contained DGS and a parasitic patch, was adopted. This technique had several advantages in the reduction in the isolation along with improvement in bandwidth, as well as gain of the antenna. Another advantage of this technique was that it neither increases the size of the antenna nor introduces any complexity. The adopted methodology reduced mutual coupling from −17.25 to −44 dB, improved impedance bandwidth from 4 to 5 GHz and gained from 9.25 to 11.9 dBi. For verification of results achieved by the software, a hardware model of the antenna was fabricated and tested, which resulted in a good comparison between both sets of values. Moreover, a comparison of the proposed MIMO antenna was carried out with the literature, which further strengthened the potential of the proposed work. The strong performance of the antenna and its MIMO configuration, backed with verified results and comparison with literary works, highlights the novelty as well as its potential for modern-day devices operating using the 28 GHz band spectrum.

## Figures and Tables

**Table 1:** Comparison between the suggested antenna and already published antenna over the same frequency.

Ref.	Dimensions (mm^3^)	Electrical Length (λ^2^)	Bandwidth (GHz)	Peak Gain (dBi)	Antenna Type
[18]	110 × 55 × 0.8	10 × 5	27–29	13	Phased Array
[19]	2 × 2 × 1.575	0.18 × 0.18	27.2–28.2	2	Meandered Radiator
[20]	6.2 × 8.4 × 1.57	0.56 × 0.76	26.2–31.8	5.06	T–shaped patch
[21]	50 × 12 × 0.787	4.54 × 1.09	26.5–29.5	11.4	Patch Array
[22]	15 × 25 × 0.203	1.36 × 2.27	26.5–29.5	5.9	Helical Inspired antenna
[23]	26.5 × 19.5 × 0.506	2.41 × 1.77	27–29	10.3	Array Antenna
[24]	5 × 5 × 0.254	0.45 × 0.45	27.5–28.5	–	Patch Antenna
[25]	150 × 75 × 0.254	13.63 × 6.81	27.5–30	–	Array Antenna
Prop.	17 × 12 × 1.52	1.54 × 1.09	26.1–31.7	8.5	Patch Antenna

**Table 2:** Overview of results offered by various design stages.

Parameters Analyzed	Bandwidth (GHz)	Coupling (dB)	Gain (dBi)
Proposed Antenna Type			
MIMO antenna	26.5–30.5	−17.25	9.25
MIMO with Parasitic patch	25.5–30.1	−26	11
MIMO with DGS	25.5–30.3	−27.5	10.9
MIMO with Parasitic patch and DGS	25.5–30.5	−44	11.9

**Table 3:** Comparison between proposed MIMO antenna design operating over 28 GHz application and work already published in the literature.

Ref.	Antenna Size (mm^3^)	Electrical Size (λ^2^)	Bandwidth (GHz)	Coupling (dB)	ECC	Methodology Adopted	Parameter Improved
[40]	48 × 31 × 0.254	4.2 × 2.7	26–31	−21	0.0015	EBG	Isolation
[41]	41 × 28 × 0.787	3.6 × 2.4	24.6–26.5	−32	0.001	Meta surface	Isolation
[42]	40 × 40 × 1.6	3.5 × 3.5	3.1–10.6	−17	0.001	DGS	Isolation
[43]	30 × 35 × 0.787	2.6 × 3	27.5–28.5	−27	0.0003	Parasitic patch	Isolation
[44]	85 × 21 × 0.508	7.4 × 1.8	27–32	−23	–	Metamaterial	Isolation
Proposed	32 × 35 × 1.52	2.8 × 3	25.6–30.5	−30	0.12	Parasitic patch + DGS	Isolation + Bandwidth + Gain

## Data Availability

All data are available in the manuscript.
